# Co-Targeting Tumor Angiogenesis and Immunosuppressive Tumor Microenvironment: A Perspective in Ethnopharmacology

**DOI:** 10.3389/fphar.2022.886198

**Published:** 2022-06-15

**Authors:** Jianbo Zhou, Li Wang, Cheng Peng, Fu Peng

**Affiliations:** ^1^ Key Laboratory of Drug-Targeting and Drug Delivery System of the Education Ministry and Sichuan Province, Sichuan Engineering Laboratory for Plant-Sourced Drug and Sichuan Research Center for Drug Precision Industrial Technology, West China School of Pharmacy, Sichuan University, Chengdu, China; ^2^ State Key Laboratory of Southwestern Chinese Medicine Resources, Chengdu University of Traditional Chinese Medicine, Chengdu, China

**Keywords:** tumor angiogenesis, targets, delivery system, immunosuppressive microenvironment, molecular intervention

## Abstract

Tumor angiogenesis is one of the most important processes of cancer deterioration *via* nurturing an immunosuppressive tumor environment (TME). Targeting tumor angiogenesis has been widely accepted as a cancer intervention approach, which is also synergistically associated with immune therapy. However, drug resistance is the biggest challenge of anti-angiogenesis therapy, which affects the outcomes of anti-angiogeneic agents, and even combined with immunotherapy. Here, emerging targets and representative candidate molecules from ethnopharmacology (including traditional Chinese medicine, TCM) have been focused, and they have been proved to regulate tumor angiogenesis. Further investigations on derivatives and delivery systems of these molecules will provide a comprehensive landscape in preclinical studies. More importantly, the molecule library of ethnopharmacology meets the viability for targeting angiogenesis and TME simultaneously, which is attributed to the pleiotropy of pro-angiogenic factors (such as VEGF) toward cancer cells, endothelial cells, and immune cells. We primarily shed light on the potentiality of ethnopharmacology against tumor angiogenesis, particularly TCM. More research studies concerning the crosstalk between angiogenesis and TME remodeling from the perspective of botanical medicine are awaited.

## Introduction

The concept of “angiogenesis switch,” first proposed by Folkman, is traced back to 1971, which depicted that the imbalance between pro-angiogenesis and anti-angiogenesis determines the survival and progression of tumors. The former includes VEGF (vascular endothelial growth factor) family, angiopoietin (Ang), platelet-derived growth factors, fibroblast growth factors (FGFs), neuropilin, transforming growth factor, insulin-like growth factor, chemokines, and semaphorins/plexins/neuropilins, while the latter is composed of endostatin, thrombospondin-1, angiostatin, and interferon-α (Zhou et al., 2017). Beyond the well-known target αvβ3 integrin (Cayrol et al., 2019), VEGF/VEGFR, FGF/FGFR, and PDGF/PDGFR axes were considered the most common signaling and pivotal role in tumor angiogenesis. The expression of numerous angiogenesis-related proteins was found in breast cancer, including VEGF, Ang-1/Tie-2, PDGF, and bFGF(FGF2) (Folkman, 2007).

**FIGURE 1:**
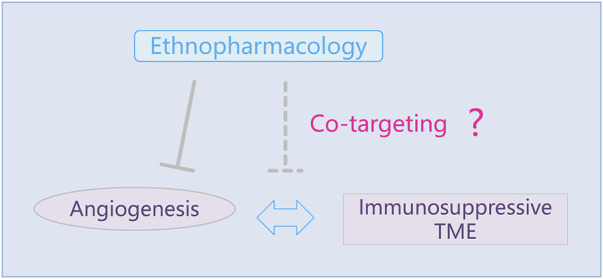
Potential and promising candidates were revealed for anti-angiogenic therapy, including traditional Chinese medicine. The attractive perspective that co-regulating angiogenesis and immunosuppressive tumor microenvironment via natural products and its derivatives or delivery system is proposed under the frame of ethnopharmacology.

Advances toward understanding anti-angiogenic therapy that blocks neo-angiogenesis and restricts nutrition and oxygen support have exerted considerable progress against cancer. More than 14 FDA-approved anti-angiogenic drugs have been applied in clinical against several cancers, which are mainly divided into two categories: small molecular tyrosine kinase inhibitors (TKIs) and monoclonal antibodies, with the representative bevacizumab (indication: colorectal, non-small-cell lung, and glioblastoma multiforme) and sorafenib (indication: renal cell and hepatocellular carcinoma), respectively (Rajabi and Mousa, 2017). According to molecular targets, anti-angiogenic drugs were composed of VEGF inhibitor, PDGF inhibitor, Ang inhibitor, and VEGFR inhibitor (Parmar and Apte, 2021). It is noteworthy that some TKIs are multi-targeting and pleiotropic. For example, sorafenib shows multi-kinase-inhibiting efficiency, including VEGFRs and PDGFR. Although anti-angiogenic therapy targeting VEGF/VEGFR prolonged the overall survival of cancer patients, these drugs lead to untoward side effects, including lethal hemoptysis and intestinal perforation (Johnson et al., 2004; Jain et al., 2006). It is reported that the absence of VEGF/VEGFR in normal endothelial cells is responsible for these adverse effects (Rini, 2007). Beyond these, drug resistance and vascular toxicity are still prominent side effects and an insurmountable challenge of anti-angiogenesis therapy (Neves et al., 2020). A cancer combination therapy that partially avoids the progression of drug resistance elevates the risk of hypertension in tumor patients (Guo et al., 2021). The phase II study provides the clinical basis for the combination of bevacizumab and trebananib (median OS 31.4 months): no increase in side effects was observed without chemotherapy (Mooi et al., 2021). After EGFR-TKI resistance, based on real-world data, there was no significant difference between chemoimmunotherapy and chemo-antiangiogenesis in the prognosis of patients with advanced non-small-cell lung cancer (Yu et al., 2021). However, the combination of drug schemes containing anti-angiogenic therapy may cause embryotoxicity, which needs to be paid attention to in pregnant female tumor patients (Al-Asmakh et al., 2021). The single-target mono-therapeutic approaches significantly evoked TKI-resistances in that molecular signaling compensation and recruitment of pro-angiogenic cells were two indispensable causes. Moreover, the phenomenon of the proportion of non-responder patients toward the anti-VEGF approach remained high. Therefore, there is an urgency to introduce new therapy to overcome these shortcomings based on anti-angiogenic therapy. Recently, the combined approach with anti-angiogenesis and immune therapy was considered a promising avenue due to it breaking the mutual support between tumor angiogenesis and immunosuppressive TME (Song et al., 2020). Worldwide traditional medicines exert alternative and supplementary roles in cancer treatment, in all of which traditional Chinese medicine (TCM) has been investigated in abundant literature. The mainstream view is that the application of TCM relieves chemotherapy- and radiotherapy-induced adverse reactions, containing gastrointestinal reactions, cardiotoxicity, and peripheral neuropathy, even acneiform eruptions and diarrhea that are EGFR-TKI related (Zhang et al., 2021a). The traditional decoction and botanical products from native medicine have been extensively observed and explored according to modern pharmacology and molecular sciences in carcinogenesis (Xiang et al., 2019). TCMs have allowed for anti-tumor effects *via* immune enhancement and perform attributes for anti-angiogenesis, respectively (Zhang et al., 2018a; Wang et al., 2020a). However, few articles have reported the anti-angiogenesis potential of native pharmacology, especially the pleiotropy of candidate molecules toward tumor angiogenesis and tumor microenvironment (TME) concurrently. Importantly, traditional and botanical medicines provide a molecule library for screening candidates against tumor angiogenesis and immunosuppressive tumor microenvironment. Thus, based on PubMed, Web of Science, and Google Scholar, this review selectively introduced dozens of representative candidate anti-angiogenic agents and enumerated several angio-active molecules from ethnopharmacology for the aforementioned combined therapy. More significantly, several candidates with multi-targeting or pleiotropic attributes performed the potential to directly realize the synergistic effects between anti-angiogenic and immune therapy (Figure 1).

## Potential Targets of Anti-angiogenesis

**FIGURE 2:**
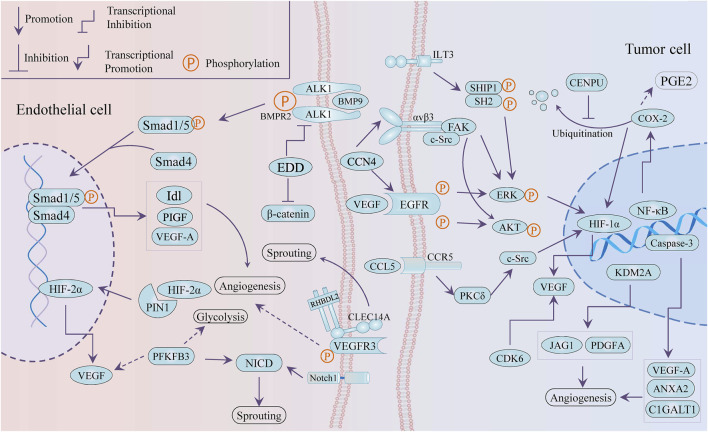
Schematic draw of potential targets against angiogenesis.

Emerging targets provide more possibilities and a greater understanding for anti-angiogenesis therapy. It is worth mentioning that tumor angiogenesis is closely associated with tumor cells releasing angiogenic factors and endothelial cells supporting vessel sprouting. As present in Figure 2, several novel targets with anti-angiogenesis nature were selectively reported, including PIN1, KDM2A, EDD, CCN4, and even glycolysis-related PFKFB3. All of these potential targets can be divided into two types: targets in vascular endothelial cells and targets in cancer cells.

### Targets in Vascular Endothelial Cells

PIN1 (peptidyl-prolyl cis–trans isomerase NIMA-interacting 1) accelerated tube formation of human umbilical vein endothelial cells (HUVECs) and angiogenesis in chick chorioallantoic membrane (CAM) by stabilizing HIF-2 α and enhancing its transcriptional activity (Choi et al., 2020). Silencing EDD (E3 isolated by differential display gene) induced the weakening of migration and tube formation of HUVECs, which was due to the negative regulation of ACVRL1 (activin receptor-like kinase-1, ALK1) gene and downstream Smad signal by EDD. In addition, transcription factor SP1 was partly responsible for the upregulation of ACVRL1 induced by blocking EDD (Chen et al., 2013a).

In addition, the type 14 family of C-type lectins have been considered promising targets for tumor treatment, including CD93, CLEC14A, and CD248 (Khan et al., 2019). C-type lectin family 14, member A (CLEC14A), overexpressed in tumor endothelial cells, promoted sprouting angiogenesis *via* VEGF/VEGFR-2/VEGFR-3 pathway (Lee et al., 2017a) and vascular development (Mura et al., 2012; Noy et al., 2016) and mediated cell–cell adhesion *via* its extracellular C-type lectin-like domain (CTLD) (Rho et al., 2011). HSP70-1A, acting as a molecular chaperone to stabilize the conformation of membrane protein CLEC14A, prompted the CLEC14A–CLEC14A interaction of endothelial cell–cell contact. These interactions triggered ERK phosphorylation and endothelial tube formation (Jang et al., 2017). The antibody deglyco C1 IgG targeting CTLD of CLEC14A was developed to inhibit CLEC14A expression and VEGF dependent angiogenesis (Kim et al., 2018).

The 6-phosphofructo-2-kinase/fructose-2,6-biphosphatase 3 (PFK-2/FBPase 3, PFKFB3), a critical regulator of glycolysis, catalyzes the conversion of fructose-6-phosphate (F6P) to fructose-1,6-bisphosphate (F1,6P2), which serves as an allosteric activator of the rate-limiting enzyme 6-phosphofructo-1-kinase (PFK-1). While PFKFB3 has been proved as an oncogene relating to cell proliferation, survival, and invasion (Shi et al., 2017; Kotowski et al., 2021), it is important to understand its role in the angiogenesis of TME, including tumor cells and vascular endothelial cells (ECs). Knockout of PFKFB3 was conducive to improving chemotherapy response and weakening tumor invasion and metastasis by normalizing tumor blood vessels, particularly converting endothelial barrier dysfunction (Cantelmo et al., 2016). On the other hand, the deficiency of PFKFB3 impaired angiogenesis involved with the modulation of tip cell formation and sprouting, while its overexpression facilitated vessel branching *via* the suppression of the pro-stalk activity that Notch signaling mediated (De Bock et al., 2013). Moreover, 3-(3-pyridinyl)-1-(4-pyridinyl)-2-propen-1-one (3PO), a molecular blocker of PFKFB3, inhibited vessel sprouting by restraining proliferation and migration of ECs and countered vascular hyper-branching that promoted inhibition of triggered Notch or VEGFR1 (Schoors et al., 2014). Another *in vivo* study found that the pro-normalization effect toward tumor blood vessels was reversed to vascular fragmentation and decomposition at high dose (70 mg/kg), compared with a low dose at 25 mg/kg. The positive correlation between PFKFB3 and CD163, CD31 suggested that PFKFB3 possibly promoted angiogenesis through modulating the infiltration of CD163 + tumor-associated macrophages (TAMs) in oral squamous cell carcinoma (Li et al., 2019a).

Galectins, cancer-associated and evolutionarily conserved glycoproteins, have been reviewed as potential targets for anti-angiogenic intervention (Varinska et al., 2017; Dings et al., 2018). Globo-H, a hexasaccharide originally found in human breast cancer cell line MCF-7, is strongly expressed in massive malignant tumors and involved in the regulation of the tumor microenvironment (Huang et al., 2020). A large number of evidences support its importance as an antigen in glycan localized tumor vaccines (Smith and Bertozzi, 2021). In the study about the cancer-associated glycans and glycosphingolipids of Globo-H ceramide (GHCer), cancer cells derived GHCer through microvesicles were assimilated by HUVECs, leading to the improvement of the angiogenic attribute, of which molecular mechanism involving the interaction between GHCer and TRAX for consequently inducing the activation of PLCβ1, drivers of early angiogenesis (Cheng et al., 2014). More investigations are expected to focus on the potential role of tumor-specific glycans and their related genes in angiogenesis. Likewise, the potential of glycoproteins in tumor angiogenesis and carcinogenesis is incomprehensively unmasked, for instance, Angiopoietin-like (ANGPTL) protein (Carbone et al., 2018).

### Targets in Cancer Cells

KDM2A was reported as a pro-angiogenesis gene in breast cancer by transactivating JAG1 and PDGFA (Chen et al., 2016). WISP-1/CCN4, an extracellular matrix-associated protein, promoted VEGF-A secretion through the integrin αvβ3/FAK/c-Src axis and the EGFR/ERK/HIF1-α signaling pathway that was transactivated subsequently, in oral squamous cell carcinoma (OSCC), and then the VEGF that CCN4 induced mediated the neovascularization of endothelial progenitor cells (EPCs) trigging (Chuang et al., 2015).

Cyclooxygenase-2 (COX-2) is closely related to cancer progression such as cancer stem cell-like activity, apoptosis, proliferation, angiogenesis, inflammation, invasion, and metastasis, which involves enormous signal pathways for which there are varieties of transcription factor-binding sites in its promoter region, including IL-1, IL-6, SP1, AP-2, NF-κB, c-Jun, and CREB (Hashemi Goradel et al., 2019). The COX-2/HIF-1a/VEGF-A axis is one of the contributors to COX-induced angiogenesis. On the other hand, COX-2 mediated arachidonic acid metabolites are conducive to tumor vascular progression. For instance, prostaglandin E2 (PGE2) participates in the production of VEGF and the improvement of sprouting, migration, and tube formation in ECs (Gately and Li, 2004). The expression of COX-2 and VEGF was inactivated by tanshinone II-A, miR-101, and andrographolide in tumor cells, while the andrographolide impaired COX-2 promoter activity and restricted multiple trans-activators to bind the COX-2 promoter, such as CREB-2, c-Fos, and NF-κB (Zhou et al., 2012a; Liu et al., 2018a; Peng et al., 2018). However, mitosis-related centromere protein U (CENPU) suppressed the ubiquitination-dependent degradation of COX-2 to maintain angiogenesis through the activation of the COX-2/p-ERK/HIF-1α/VEGFA signaling axis (Pan et al., 2020).

Not only angiogenesis but some targets also interfere with cancer phenotypes, such as proliferation, apoptosis, and even metabolic reprograming, but in the context the TME regulation is our interest. ILT3, an immune negative regulator in non-solid tumors, potentiated tumor metastasis and angiogenesis in non-small-cell lung cells (NSCLCs). ILT3 recruited SHP2 and SHIP1, followed by phosphorylation of ERK1/2 to induce angiogenesis with increased VEGF-A expression (Li et al., 2021a). CCL5/CCR5 signaling mediates signal transduction cascades related to tumor progression, including PI3K/Akt, JAK/STAT3, MAPK/ERK, and NF-kB, involving in tumor growth, metastasis, cancer stem cell expansion, DNA damage repair, and angiogenesis and metabolic reprograming. It is noteworthy that the axis recruits immune cells and induces immunosuppressive polarization of macrophages to modulate TME reprogramming (Aldinucci et al., 2020). Blocking the CCL5/CCR5 axis induced decreased endothelial cell migration, which was related to decreased activity of the mTOR /Akt pathway, while CCL5 promoted tumor angiogenesis through the PKC δ/c-Src/HIF-1 α/VEGF signaling pathway (Wang et al., 2015a; Sax et al., 2016). PLD1 deficiency triggered a decrease in tumor growth and angiogenesis in the xenograft model and also reduced endothelial cell adhesion by downregulating the phosphorylation of ERK 1/2, p38, and Akt (Chen et al., 2012). In addition to the involvement of apoptosis through the downregulation of pro-apoptotic genes, caspase-3 was found to play an important role in angiogenesis by transactivating pro-angiogenetic genes (VEGFA, ANXA2, and C1GALT1), showing the crosstalk between apoptosis and angiogenesis (Bernard et al., 2019). CDK6, as a component of the transcription complex, induced the expression of P16 and VEGF-A, which bridged the cell cycle and angiogenesis (Kollmann et al., 2013). A C-glycosyl flavone was reported to induce apoptosis, cell cycle arrest, and angiogenesis inhibition *via* modulating CDK6, which was consistent with CDK6 blocker (Bevara et al., 2018).

The endocannabinoid system (ECS) was associated with declined angiogenesis *via* downregulating the VEGF/PIGF/Ang-2signaling axis that was mediated by cannabinoid receptors CB1R/CB2R, and it was important that ECS induced TME remodeling for the cannabinoid receptors expressed extensively (Iozzo et al., 2021). The aforementioned effects of proteoglycan agrin remained VEGFR2 dependent (Chakraborty et al., 2020). In addition, perlecan/HSPG2, a heparan sulfate proteoglycan, gathered in the tumor marginal stromal, was considered a molecular switch of angiogenesis in TME (Cruz et al., 2020). The immune checkpoint B7-H3 (CD276) accelerated immunosuppression of TME and exhibited non-immunological attributes for participating in angiogenesis (Feng et al., 2021). In triple-negative breast cancer, targeting B7-H3 led to vessel normalization and consequently improved PD-1 treatment response (Cheng et al., 2021). Moreover, CD276 enhanced the angiogenic function of tumor-associated macrophages, and CD276-blocking antibody raised the therapeutic efficiency of paclitaxel /anti-PD-1 in 4T1 tumor-bearing mice (Cheng et al., 2021). Diversely, the potential role of semaphorin 4D (SEMA4D, CD100), glypican-1, Delta-like 1 (DLL1), and insulin-like growth factor (IGF) in the bi-directional dialog of angiogenesis and immune regulation is also of significance (Lee et al., 2015; Wu et al., 2016; Lund et al., 2020; Zhang et al., 2021b).

## The Role of Ethnopharmacology in Tumor Angiogenesis

**FIGURE 3:**
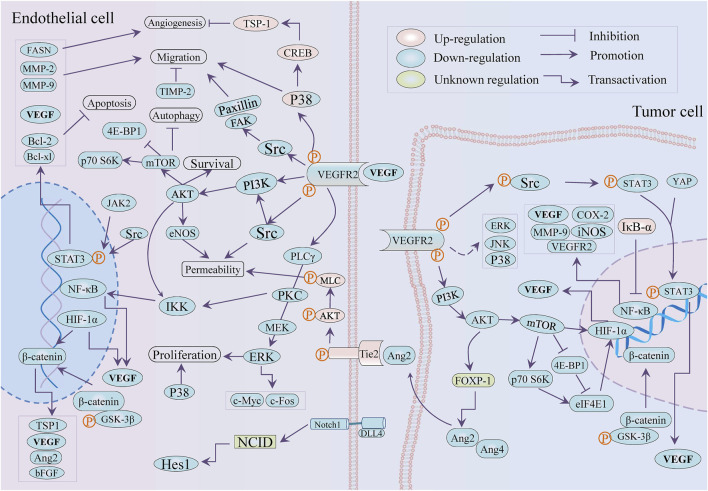
Schematic role of ethnopharmacology in tumor angiogenesis.

Natural products or phytochemicals allow for the activity of regulating angiogenesis, which has attracted extensive interest. Some pro-angiogenic molecules have been reported that the notoginsenoside Ft1 induced angiogenesis by activating the VEGF/VEGFR2 signaling, while notoginsenoside R1 activated the Ang2/Tie2 signaling pathway (Shen et al., 2012; Zhong et al., 2020). Xue et al. reported six potential tumor angiogenic inhibitors from Chinese botanical medicine (glycopeptides, flavonoids, artemisinin, arsenic trioxide, ginsenoside, and tanshinone) and their pharmacological mechanisms (Yang and Wu, 2015). As shown in Table 1 and Figure 3, we illuminated the potential of plant-derived components and traditional Chinese medicine for anti-angiogenesis therapy: the former mainly included artemisinin, tanshinone, flavonoids, and saponin, while the latter is mainly composed of decoction of traditional Chinese medicine.

**TABLE 1:** Anti-angiogenic effects and mechanisms of representative molecules from ethnopharmacology.

Molecule	Model	Effect	Main mechanism	Reference
**Plant-derived molecules**				
NLGP	Swiss and C57BL/6 mice	Normalization of tumor vasculature	CD31, VEGF, and VEGFR2 ↓; CD8^+^ cell ↑	Banerjee et al. (2014)
NLGP	B16F10 cells and C57BL/6J mice	Decreasing VEGF	p-STAT3 and HIF-1α ↓	Saha et al. (2020)
*Dolichos lablab* L. lectin (DLL)	HUVECs; CAM, Rat aortic ring assay, and mice with solid lymphoma or ascites tumor	Tube formation inhibiting; declining angiogenesis *ex vivo* and *in vivo*	NF‐κB, VEGF, MMP-2, and MMP-9	Vigneshwaran et al. (2017)
Artemisinin	HUVECs with CM from osteosarcoma cell lines MG-63, U2OS; xenografts mice bearing tumors	Inhibiting migration and tube formation; reducing MVD	p38 MAPK/CREB/TSP-1 ↑	Li et al. (2019b)
Artemisinin	HUVECs; Xenografts mice bearing mda-mb-231 cells, CAM, Matrigel plug assay, and rat aortic ring assay	Inhibiting tube formation and migration; reducing MVD *in vivo* and *ex vivo*	CREB/VEGF in cancer ↓; FAK, AKT, ERK, p38, and eNOS in HUVECs↓	Tsui et al. (2019)
Dihydroartemisinin	HUVECs	Suppressing tube formation, proliferation; inducing autophagy	p-STAT3, FASN ↓, ERK1/2, c-Fos, and c-Myc ↓; LC3-II↑, phosphorylation of Akt, mTOR, p70S6K, and 4E-BP1 ↓	(Dong et al., 2015; Liu et al., 2019; Gao et al., 2020)
Tanshinone-1	HMEC-1 cells, CAM, and aortic ring sprouting assay; breast cancer MCF-7 cells	Inhibiting proliferation, tube formation, migration, and angiogenesis; reducing secretion of VEGF	HIF-1α and p-705-Stat3 in endothelial and cancer cells ↓	Wang et al. (2015b)
Tanshinone IIA	HUVECs and CAM; colorectal cancer HT-29 cells and tumor nude mice bearing the cells	Declining tube formation and angiogenesis; decreasing VEGF, bFGF secretion, and MVD	TGF-β1 or HIF-1 mediating β-catenin/TCF3/LEF1 pathway ↓	Sui et al. (2017)
Tanshinone IIA	Endothelial progenitor cells; CAM and Matrigel plug assay	Inhibition of migration and tube formation; reduction of angiogenesis	Phosphorylation of PLC and Akt and JNK ↓	Lee et al. (2017b)
Tanshinone IIA	HUVECs, CAM, and rat aortic ring assay	Suppressing proliferation, migration, tube formation, and angiogenesis	VEGFR2, CD146, and MMP-2,9 ↓	Xing et al. (2015)
Tanshinone IIA	HUVECs and CAM	Decreasing tube formation, invasion, and angiogenesis	MMP-2 ↓; TIMP-2 ↑	Tsai et al. (2011)
Tanshinone IIA	HUVECs; colorectal cancer HCT116 cells	Inhibiting proliferation, tube formation, and migration; decreasing VEGF and bFGF	HIF-1α in cancer ↓	Zhou et al. (2020a)
Tanshinone IIA	HUVECs; BALB/c nude mice with HT-29 colorectal tumor	Promoting migration and declining permeability of epithelial cells; normalization of tumor vessels	Ang2↓; Tie2-AKT-MLCK pathway ↑	Zou et al. (2021)
Tanshinone IIA	Breast cancer MCF-7 and MDA-MB-231 cells; MDA-MB-231 xenograft nude mice	Inhibition of HIF-1α and angiogenesis *in vivo*	mTOR/p70S6K/4E-BP1 signaling ↓	Li et al. (2015)
Silibinin	Cervical HeLa cells and hepatoma Hep3B cells	Decreasing HIF-1α and VEGF	mTOR/p70S6K/4E-BP1 signaling ↓; p-Akt ↑	García-Maceira and Mateo, (2009)
Imperatorin	HCT116 and its xenograft nude mice	Inhibiting HIF-1α *in vivo* and *in vitro*; reducing MVD and VEGF *in vivo*	mTOR/p70S6K/4E-BP1 signaling ↓; p-ERK and pJNK and p-p38 ↓	Mi et al. (2017)
Cryptotanshinone	HUVECs	Suppression of migration, invasion, and tube formation	VEGF, cyclin D1, β-catenin ↓; VEGFR2 pathways (p-VEGFR2, p-ERK1/2, p-p90RSK, p-Src, and p-FAK)↓	(Chen et al., 2014; Xu et al., 2017)
Cryptotanshinone	HUVECs and aortic ring sprouting assay	Inhibition of CT26 cell-stimulated tube formation and vessel sprouting *ex vivo*	VEGF, CD31, CD34, VEGFR2, and HIF-1α ↓; PI3K/Akt/mTOR signaling in CT26 cells ↓	Zhang et al. (2018b)
Silybin A, silybin B, isosilybin A, and isosilybin B	HUVECs, aortic ring sprouting assay; prostate cancer DU145 xenograft mice	Inhibition of VEGF-induced proliferation, tube formation, migration, and vessel sprouting; downregulating VEGFR1, HIF-1α, and Akt in xenografts	Akt/HIF-1 α/VEGF axis in prostate cancer ↓; VEGFR2 and its downstream Akt/MAPKs/mTOR axis ↓	Deep et al. (2012)
Silibinin	Human endothelial ECV304 cells	Induction of apoptosis	Bcl-2, P65 ↓; cytochrome c release and cleavage of caspase-3, caspase-9, and PARP ↑	Yoo et al. (2004)
Silibinin	HUVECs	Induction of cell cycle arrest, apoptosis, and suppression of migration and tube formation	Survivin, Akt, and NF-κB ↓	Singh et al. (2005)
Silibinin	A/J mice with azoxymethane-induced colon cancer	Decreasing VEGF *in vivo*	IGFBP-3 ↑; β-catenin, IGF-1Rβ, pGSK-3β, and pAkt ↓	Ravichandran et al. (2010)
Silibinin	HT-29 cells xenograft mice	Reducing MVD	NOS, COX-2, HIF-1α, VEGF, Ang-2, and Ang-4 ↓	Singh et al. (2008)
Silibinin	Transgenic mouse model of prostate cancer	Reducing MVD	VEGF, VEGFR, HIF-1α, and iNOS ↓	Raina et al. (2008)
Dioscin	C57BL/6 mice bearing B16F10 melanomas; CAM; HUVECs with A375 cells CM	Reducing MVD; decreasing angiogenesis; inhibiting tube formation	p-Src/p-STAT3/VEGF/MMP-2,9 in melanoma ↓	Liu et al. (2022)
Dioscin	HUVECs; Matrigel plugs assay; colon cancer C-26 cells xenograft mice	Inhibiting proliferation, migration, invasion, and tube formation; reducing angiogenesis *in vivo*	VEGFR2 and Akt/MAPK signaling pathway ↓	Tong et al. (2014)
Moscatilin	HUVECs; Matrigel plugs assay; lung cancer A549 cells xenograft mice	Suppressing proliferation, migration, and tube formation; reducing angiogenesis *in vivo*	p-ERK1/2, p-Akt, and p-eNOS ↓	Tsai et al. (2010)
Luteolin	Vascular endothelial cells of NSCLC	Suppressing proliferation, migration, and invasion	VEGF, MMP-2, MMP-9, PURB, and PI3K/Akt/MAPK axis ↓; miR-133a-3p ↑	Pan et al. (2022)
Luteolin	HUVECs; rabbit corneal neovascularization assay and A-431 murine xenograft model	Inhibiting proliferation and survival; decreasing angiogenesis	PI3K/Akt/p70 S6K ↓	Bagli et al. (2004)
Luteolin	HMEC-1; aortic ring sprouting assay and CAM	Inhibiting proliferation, migration, invasion, and tube formation; reducing microvessel sprouting and angiogenesis	Gas6/Axl-mediated PI3K/Akt/mTOR axis ↓	Li et al. (2017)
Luteolin	HUVECs; melanoma cells A375 and B16F10	Inhibiting tube formation; suppressing HIF-1α /VEGF expression	p-Akt and p-VEGFR-2 in cancer ↓	Li et al. (2019c)
Timosaponin AIII	HUVECs; transgenic zebrafish	Inhibiting proliferation, migration, invasion, and tube formation; reducing intersegmental vessels and subintestinal vessels	VEGFR2/PI3K/Akt/MAPK signaling pathway ↓	Zhou et al. (2020c)
Paris saponin I	HUVECs	Inhibiting proliferation, migration, invasion, and tube formation; inducing apoptosis and cell cycle arrest	VEGFR2/PI3K/Akt/MAPK, Src/eNOS, PLCγ/MEK/ERK, and JAK2/STAT3 ↓	Wang et al. (2020b)
Polyphyllin VII	HUVECs; Zebrafish embryo assay	Inhibiting viability, migration, invasion, and tube formation; reducing angiogenesis	NF-κB/MMP-9/VEGF pathway in HCC cells	Zhang et al. (2021c)
Farrerol	HUVECs	Inhibiting proliferation, migration, invasion, and tube formation; inducing apoptosis and cell cycle arrest	Erk, Akt, mTOR, Jak2, STAT3, Bcl-2, and Bcl-xl ↓	Dai et al. (2016)
Umbelliprenin	Breast cancer cells 4T1 tumor-bearing balb/c mice	Reducing tumor angiogenesis	VEGF, CD31, MMP2, MMP9, VCAM1, and NF-κb ↓	Rashidi et al. (2018)
Gambogic acid	Myeloma U266 cells; U266 xenograft mouse model	Reducing tumor angiogenesis	Akt/mTOR/HIF-1α/VEGF ↓	Wang et al. (2014)
Gambogic acid	Rat aortic ring assay, CAM, and C57BL/6 mice bearing lung cancer; HUVECs	Reducing angiogenesis *in vivo* and *in vitro*; inhibiting migration and tube formation	Phosphorylation of VEGFR2, ERK1/2, Akt, and p38 MAPK ↓	Lu et al. (2007)
Gambogic acid	C57BL/6 mice with B16F10 melanoma or MC38 colon cell, CAM, Aortic ring assay, Spheroid sprouting assay; HUVECs	Reducing angiogenesis *in vivo* and *ex vitro*; inhibiting proliferation and migration	YAP and p-STAT3 ↓	Wan et al. (2019)
**Traditional Chinese medicine against tumor angiogenesis**				
Arsenic trioxide (As2O3)	HUVECs; NCI-H69 cells xenograft mice	Suppressing tube formation; decreasing MVD	Dll4, Notch1, and Hes1 ↓	Yang et al. (2019a)
Ethanolic extract of *Artemisia sieberi*	HUVECs and CAM	Inhibition of tube formation and angiogenesis	VEGFR-1, VEGFR-2, and CD34 in transcript ↓	Abdolmaleki et al. (2016)
Ethanol extract of *Amomum tsaoko*	HUVECs with ovarian cancer SKOV3 CM; BALB/c nude mice bearing SKOV3 tumor	Inhibiting migration, invasion, and tube formation; reducing MVD	p-STAT3, NF-kB, IL-6, and VEGF ↓	Chen et al. (2020a)
Aqueous extract of Yu Ping Feng San decoction	HUVECs; orthotopic murine transplanted model of HCC	Inhibiting proliferation and migration; reducing MVD	VEGF, TSLP, and p-STAT3 ↓	Yuan et al. (2019)
Aqueous extract of Shiquan Yuzhen decoction	Murine xenograft model of Lewis	Reducing MVD	VEGFA, HIF-1α↓; CD8^+^ T, and Treg cells ↑	Sun et al. (2021)
Aqueous extract of Xiaotan Sanjie decoction	Murine xenograft model of gastric cancer; HUVECs co-cultured with gastric cancer SGC-7901 cells	Reducing MVD; inhibiting migration and tube formation	Notch-1, Hes1, VEGF, and VEGFR1/2 ↓	(Yan et al., 2014; Shi et al., 2016)
Ethanol extract of Jiedu recipe	Endothelial EA.hy 926 cells; HCC Huh 7 cells	Inhibiting proliferation and tube formation	VEGFR, p-Akt, p-Erk, p- NF-kB, and HIF-1α in cancer ↓	Lin et al. (2021)

HUVECs, human umbilical vascular endothelial cells; CAM, chick chorioallantoic membrane; MVD, microvascular density; HMEC, human microvascular endothelial; mTOR, mammalian target of rapamycin; p70S6K, ribosomal protein S6 kinase; 4E-BP1, eukaryotic initiation factor 4E-binding protein-1; CM, conditioned media; NSCLC, non-small-cell lung cancer; HCC, hepatocellular carcinoma.

### Plant-Derived Molecules Against Angiogenesis

MicroRNAs played a crucial role in ginsenoside-mediated anti-angiogenesis (Ashrafizadeh et al., 2020). For instance, the ginsenoside Rg1 downregulated miR15-b to induce angiogenesis in an increased VEGFR-2 manner (Chan et al., 2013). Notwithstanding the double-edged sword role of ginsenoside Rg3 in tumor angiogenesis is worth discussing, it was broadly considered an anti-angiogenic agent by modulating pro-angiogenic factors VEGF, FGF, and MMP (Nakhjavani et al., 2020; Liu et al., 2021a). Most recently, Rg3 was reported to induce angiogenesis inhibition in precancerous lesions of gastric cancer through lessening GLUT1 and GLUT4 (Zeng et al., 2022). Interestingly, the optimized combination of Rg3 epimers (50 μM *S*-Rg3 + 25 μM *R*-Rg3) more effectively suppressed tube formation, migration, and proliferation of HUVECs than *S*-Rg3 and *R*-Rg3, respectively (Nakhjavani et al., 2021). Although several ginsenosides active components of ginseng, including Rb1, Rg3, and Rd served as tumor angiogenesis inhibitors (Li et al., 2021b), it is noteworthy that the angiogenic modulation of the rest of the ginsenosides, containing protopanaxadiols, protopanaxatriols, and oleanane types (Zhou et al., 2022), were incompletely investigated. In addition, neem leaf glycoprotein (NLGP), a natural immune-modulator obtained from the leaves of neem (*Azadirachta indica* A. juss), balked M2 polarization of TAMs and HIF1α/VEGF signaling with STAT3-dependent manner and induced tumor vessel normalization with CD8^+^ T cells dependence by downmodulating VEGF and VEGFR2 (Banerjee et al., 2014; Goswami et al., 2014; Saha et al., 2020). As a part of the anti-tumor mechanism of legume lectin proteins, its anti-angiogenesis effect has been reported that *Dolichos lablab* L. lectin (DLL) protein weakened the expression of pro-angiogenic factors encompassing NF-κB, HIF-1 α, MMP-2 and 9, and VEGF, while the concanavalin A exhibited anti-angiogenic action *via* targeting IKK-NF-κB-COX-2, SHP-2-MEK-1-ERK, and SHP-2-Ras-ERK cascade (Li et al., 2011; Vigneshwaran et al., 2017). Another study on Lectin from *Laetiporus sulphureus* (LSL) revealed the LSL effects of anti-angiogenesis in zebrafish and migration inhibition in endothelial cells (Petrović et al., 2020).

Beyond antimalarial drugs, traditional Chinese medicine-derived artemisinin (ART) and its derivatives have attracted emerging concern for the promising potential in cancer therapy (Crespo-Ortiz and Wei, 2012; Li et al., 2020a). Tianshu et al. implied that attributes of artemisinin and its analogs against angiogenesis were associated with PI3K/Akt/mTOR axis, JNK, and p38 MAPK (Wei and Liu, 2017). Dissimilarly, artemisinin facilitated TSP-1 release to inhibit osteosarcoma-induced angiogenesis by activating the phosphorylation of p38 MAPK/CREB (Li et al., 2019b). In HUVECs, dihydroartemisinin (DHA) impaired proliferation and loop formation by inhibiting ERK signaling, p-STAT3 and its downstream fatty acid synthase (FASN) expression, and triggered autophagy *via* Akt/mTOR pathway (Dong et al., 2015; Liu et al., 2019; Gao et al., 2020). DHA attenuated HUVECs-mediated angiogenesis by modulating IκB-α/NF-κB/VEGFR2 axis (Dong et al., 2014), while DHA promoted VEGFR1 expression *via* upregulating ETS-1 (Dong et al., 2014; Niu et al., 2018). Unexpectedly, the ethanolic extract of *Artemisia sieberi* Besser performed stronger antiangiogeneic properties in tube formation and CAM assay in contrast to ART, which was attributed to the discordantly reduced VEGFR-1, VEGFR-2, and CD34 in the transcript (Abdolmaleki et al., 2016).

Tanshinone IIA (Tan IIA) is one of the main active components of Salviae miltiorrhizae radix et rhizome (*Salvia miltiorrhiza* Bunge) and is famous for its effectiveness in the treatment of cardiovascular diseases (Guo et al., 2020; Zhong et al., 2021). Its broad-spectrum pharmacological activities include but are not limited to anti-tumor, while there are few literatures concerning angiogenesis in tumors. Tan IIA suppressed β-catenin/TCF3/LEF1/VEGF by TGF-β1 at normoxia while by HIF-1α at hypoxia to astrict angiogenesis in colorectal cancer (Sui et al., 2017). Regardless of at hypoxia or at normoxia, tanshinone I subdued angiogenesis in epithelial cells (HMEC-1) and the secretion of VEGF from tumor cells (MCF-7) by the common mechanism: deduction of p-STAT3 and HIF-1α, and also inhibited VEGF against lung carcino-angiogenesis (Tung et al., 2013; Wang et al., 2015b). The angiogenesis EPCs-mediated was diminished by tanshinone IIA *in vitro* and *in vivo* by ruling the VEGF/PLC/Akt/JNK signaling axis (Lee et al., 2017b). The VEGF/VEGFR2 pathway and MMP-2/-9 and TIMP-2 expression were downregulated by Tan IIA in HUVECs for countering angiogenesis, while Tan IIA could bind the VEGFR2 kinase domain to inhibit the VEGF/VEGFR axis in lung cancer A549 cells (Tsai et al., 2011; Xie et al., 2015; Xing et al., 2015). Tan IIA also repressed the expression of pro-angiogenic factors (VEGF and bFGF) and HIF-1α in colorectal cancer (CRC) HCT-116 cells and adversely regulated proliferation and tube formation of HUVECs (Zhou et al., 2020a). Moreover, Tan IIA opposed angiogenesis with COX-2 and VEGF dependent in mice xenograft model of CRC and ovarian cancer (Zhou et al., 2012b; Zhou et al., 2020b). The mTOR/p70S6K/4E-BP1 and MAPK signaling pathway involved the anti-angiogenic activity that induced VEGF/HIF-1α suppression of silibinin and imperatorin in cervical and hepatoma cancer cells and colon cancer, respectively (García-Maceira and Mateo, 2009; Mi et al., 2017). Uniformly, Tan IIA weakened VEGF/HIF-1α expression by controlling the mTOR /p70S6K /RPS6 /4E-BP1 axis in breast cancer (Li et al., 2015). It was of importance that Tan IIA caused vascular stability and vascular normalization *via* downregulating Ang2-Tie2-AKT-MLCK axis in colon cancer (Zou et al., 2021). The anti-angiogenic properties of tanshinone VI were attributed to its downregulation of adhesion molecules ICAM-1 and VCAM-1 in epithelial cells (Nicolin et al., 2013). Moreover, the angiogenesis inhibition of cryptotanshinone (CPT) was implicated in multi-signaling, including Wnt/β-catenin/VEGF axis, VEGFR2 and its downstream Src/FAK, ERK1/2 in HUVECs, while it leads to the downregulation of PI3K/Akt/mTOR signaling and HIF-1α in CT26 colon cancer cells (Chen et al., 2014; Xu et al., 2017; Zhang et al., 2018b).

The silymarin, silibinin (SB), and thalidomide attenuated proliferation in endothelial (EA.hy 926) and colon cancer (LoVo) cell lines and also reduced the LoVo-secreting VEGF (Yang et al., 2003). The two pairs of flavonolignan diastereoisomers (silybin A, silybin B, isosilybin A and isosilybin B) isolated from *Silybum marianum* (L.) exerted similar effectivity against angiogenesis *via* downregulating Akt/HIF-1 α/VEGF axis in prostate cancer, and simultaneously modulated VEGF-induced signaling, encompassing VEGFR and its downstream Src, Akt, MAPKs, mTOR and so on in HUVECs (Deep et al., 2012). Hyeon et al. deemed that the anti-angiogenic effect of silibinin in endothelial cells depended on the regulation of NF-κB and apoptosis induction with the Bcl-2 family and Caspases involved (Yoo et al., 2004). Rana et al. found SB-induced cell cycle arrest, apoptosis, and suppression of migration and tube formation to perform anti-angiogenic efficacy in HUVECs, with survivin, Akt, and NF-κB were decreased (Singh et al., 2005). In addition, it reduced iNOS, COX-2, and VEGF expression in colon cancer mice, with the decreased levels of β-catenin, IGF-1Rβ, p-GSK-3β and p-Akt, and enhanced expression of IGFBP-3 (Ravichandran et al., 2010). Research on colon cancer suggested that the downregulation of NOS, COX-2, HIF-1α, VEGF, Ang-2, and Ang-4 was the result of SB treatment (Singh et al., 2008; Sameri et al., 2021). The SB lessened tumor angiogenesis in pancreatic cancer and prostate tumor xenograft (Singh et al., 2003; Nambiar et al., 2013), while SB inhibited tumor angiogenesis *via* restricting VEGF, VEGFR2, HIF-1α, and iNOS expression in a transgenic mouse of prostate cancer (Raina et al., 2008).

Luteolin’s effect against angiogenesis in vascular endothelial cells was ascribed to multiple mechanisms, such as the inhibition of MAPK and PI3K/Akt pathways that miR-133a-3p/PURB- mediated, repression of the PI3K/Akt/p70 S6K signaling and Gas6/Axl axis (Bagli et al., 2004; Zhu et al., 2013; Li et al., 2017; Pan et al., 2022). In addition, luteolin impaired HIF-1α/VEGF and Notch1-VEGF signaling in melanoma and gastric cancer individually (Zang et al., 2017; Li et al., 2019c). The luteolin showed better attributes in the suppression of blood vessels in CAM assay, cell proliferation and cell migration assay in HT-29 cells than lupeol and lectin (Ambasta et al., 2015). HIF-1 α was considered as a pro-angiogenic factor to facilitate tumor angiogenesis by activating PI3K/MAPK pathway and inducing VEGF release. The combination of asparagus polysaccharide (IC_50_ ∼ 10 mg/ml) and HIF-1α RNAi significantly inhibited the tube formation in HUVECs under HCC cells (SK-HEP and HEP-3B) induced and tumor angiogenesis in a xenotransplantation mouse model (100 mg/kg by gavage), and reduced the expression of VEGF and HIF-1α by suppressing Akt/Erk axis *in vivo* and *in vitro* (Zhu et al., 2021a).

Xanthomicrol, a flavone extracted from *Dracocephalum kotschyi* Boiss leaf, showed an antiangiogeneic effect in mice melanoma (B16F10) model (50 mg/kg) through negatively regulating the expression of VEGF, HIF-1α, and p-Akt (Ghazizadeh et al., 2020). Curcumin downregulated NF-κB and FAK/P38 MAPK and reduced the expression of VEGF, MMP-2, MMP-9, and COX-2 to exert the anti-tumor angiogenesis attribute *in vivo* and *in vitro* (Kumar et al., 2016; Hosseini et al., 2019). Compared with curcumin, bisdemethoxycurcumin is more effective to downregulate angiogenetic makers NF-κB, COX-2, MMP-9, and VEGF in Hep-2 cells (Mohankumar et al., 2021). However, curcumin promoted endothelial progenitor cells (EPCs) to participate in angiogenesis and conduced to neovascularization in animal models *in vivo* (Wang and Chen, 2019). In addition, VEGF-A and COX2 mRNA was downregulated by umbelliprenin (UMB, a coumarin from Ferula species) in 4T1 tumor mice (2.5 mg/d), with the protein expression of NF-κB and VCAM1 decreasing (Rashidi et al., 2018). The prior review has depicted the anti-angiogenic function of gambogic acid (GA) against tumors depending on the obstruction of HIF-1α/VEGF and prolyl hydroxylase-2 (PHD2)–von Hippel-Lindau gene (VHL)–HIF-1α, along with EGFR2 pathway (Liu et al., 2020). Moreover, GA mediated the inhibition of HIF-1α/VEGF through the downregulation of PI3K/Akt/mTOR in myeloma cells (Wang et al., 2014). After GA treatment, YAP/p-STAT3 and phosphorylation of VEGFR2 (KDR/Flk-1) signaling axis was suppressed in HUVECs (Lu et al., 2007; Wan et al., 2019).

The antiangiogeneic effect of Dioscin, a steroid saponin mainly appearing in *Dioscorea opposita* Thunb, involved the downregulation of p-Src/p-STAT3/VEGF/MMP-2,9 in melanoma, and attenuated VEGFR2 and Akt/MAPK signaling axis in colon cancer, while led to the constraint of VEGF/VEGFR pathway in ovarian cancer cells (Tong et al., 2014; Guo and Ding, 2018; Liu et al., 2022). Similar in mechanism, moscatilin, a bibenzyl derivative isolated from TCM Orchidaceae Dendrobii Caulis (*Dendrobium loddigesii* Rolfe), exerted antiangiogeneic attribute *in vitro via* repressing ERK1/2, Akt, and eNOS axis in HUVECs (Tsai et al., 2010). Anemarrhena saponin AIII, extracted from *Anemarrhena asphodeloides* Bunge a traditional Chinese medicine, inhibited the formation of internode vessels and subintestinal vessels in zebrafish (0.five to two µM), and significantly decreased the activity (more than 4 uM), migration, invasion and tube formation of HUVEC cells (0.5–4 µM) through attenuating VEGF/PI3K/Akt/MAPK signal transduction (Zhou et al., 2020c). Also, in HUVECs, Farrerol, a natural flavonoid from *Rhododendron dauricum* L. exerted similar mechanisms against angiogenesis by downregulating Akt/mTOR, ERK and JAK2/STAT3 signal pathway (Dai et al., 2016). Paris saponins I (polyphyllin D), existing in the Chinese herb *Paris polyphylla* var. *yunnanensis*, showed excellent anti-angiogenesis on HUVEC cells through downregulation of VEGFR2, JAK2/STAT3 pathways, and VEGFR2 and its downstream PI3K/Akt/MAPK, Src/eNOS, and PLCγ/ERK/MERK (Wang et al., 2020b). The role of Polyphyllin VII and Paris saponin II (formosanin C) against tumor angiogenesis involved NF-κB/VEGF axis on Hepatocellular carcinoma cells and ovarian cancer cells, respectively (Yang et al., 2015; Zhang et al., 2021c).

### Traditional Chinese Medicine Against Tumor Angiogenesis

As_2_O_3_, a toxic traditional Chinese medicine, inhibited tumor growth and microvessel density by downregulating Notch pathway-related proteins Hes1, Dll4, and Notch1 in the small-cell lung cancer (SCLC) mouse model (2.5 and 5 mg/kg), and As_2_O_3_ restrained with the tube-forming ability of endothelial cells through the expression of Notch 1 and Hes1 in HUVECs (Yang et al., 2019a). Indeed, the thymic stromal lymphopoietin (TSLP) protein elicited immune-suppressive TME *via* interacting with TSLP receptor in CD4^+^ T cells to promote production of immunosuppressive factors, including IL-10 and IL-13 (Sims et al., 2000). Yu Ping Feng San, a famous decoction in TCM and comprised Astragali Radix (Huangqi, the root of Astragalus membranaceus (Fisch.) Bunge or Astragalus membranaceus (Fisch.) Bunge var. mongholicus (Bunge) P. K. Hsiao), Atractylodis Macrocephalae Rhizoma (Baizhu, the rhizomes of *Atractylodes macrocephala* Koidz.), and Saposhnikoviae Radix (Fangfeng, the roots of Saposhnikovia divaricata (Turcz.) Schischk.) reduced MVD and VEGF *via* downregulation of the TSLP /STAT3 pathway in hepatocellular carcinoma and HUVECs (Yuan et al., 2019; Du et al., 2021). The ethanol extract of Amomi Fructus (the fruit of *Amomum villosum* Lour.) had no influence on the viability of vascular endothelial cells. But it inhibited angiogenesis by restricting the p-STAT3 and NF-κB expression and reducing IL-6 and VEGF secreted by ovarian cancer cells (Chen et al., 2020a). Freshly, Shiquan Yuzhen Decoction, consisting of Ginseng Radix et Rhizoma, Astragalus membranaceus (root of Astragalus membranaceus (Fisch.) Bge. var. mongholicus (Bge.) Hsiao or A. membranaceus (Fisch.) Bge.), Dioscoreae Rhizoma (rhizome of *Dioscorea opposita* Thunb.), Anemarrhenae Rhizoma (rhizome of *Anemarrhena asphodeloides* Bge.), radix scrophulariae (root of *Scrophularia ningpoensis* Hemsl.), Os Draconis, Ostreae Concha, Salviae Miltiorrhizae Radix et Rhizoma (root and rhizome of *Salvia miltiorrhiza* Bge.), and Curcuma zedoariae (rhizome of *Curcuma phaeocaulis* Val. or *C. kwangsiensis* S. G. Lee et C. F. Liang or *C. wenyujin* Y. H. Chen et C. Ling), triggered the inhibition of tumor angiogenesis *via* restricting HIF-1α/VEGFA release, recuperated the immunity with the enhancement of CD8^+^ T and Treg cells, TNF-α level, and the abatement of IL-6 in lung cancer-bearing mice (Sun et al., 2021). The attenuated angiogenesis mediated by Xiaotan Sanjie decoction that comprised eleven herbs (including *Pinelliae rhizome*, *Rhizoma arisaematis*, and *Poria cocos*) in gastric cancer was related to the Notch-1/VEGF and IL-8/VEGF/VEGFR signaling axis (Yan et al., 2014; Shi et al., 2016). Jiedu Recipe, consisting of Pleiones Pseudobulbus (pseudobulb of *Cremastra appendiculata* (D. Don) Makino or *Pleione bulbocodioides* (Franch.) Rolfe or P. yunnanensis Rolfe), valvate actinidia (root of *Actinidia valvata* Dunn.), *suppressed* hypoxia-induced angiogenesis *via* restricting IL-8/HIF-1α/PI3K and MAPK/ERK pathways in endothelial EA.hy 926 cells, and inhibited the expression of VEGF, HIF-1α, and IL-8 under hypoxic conditions in HCC Huh-7 cells (Lin et al., 2021).

## The Analogs From Medical Chemistry

**FIGURE 4:**
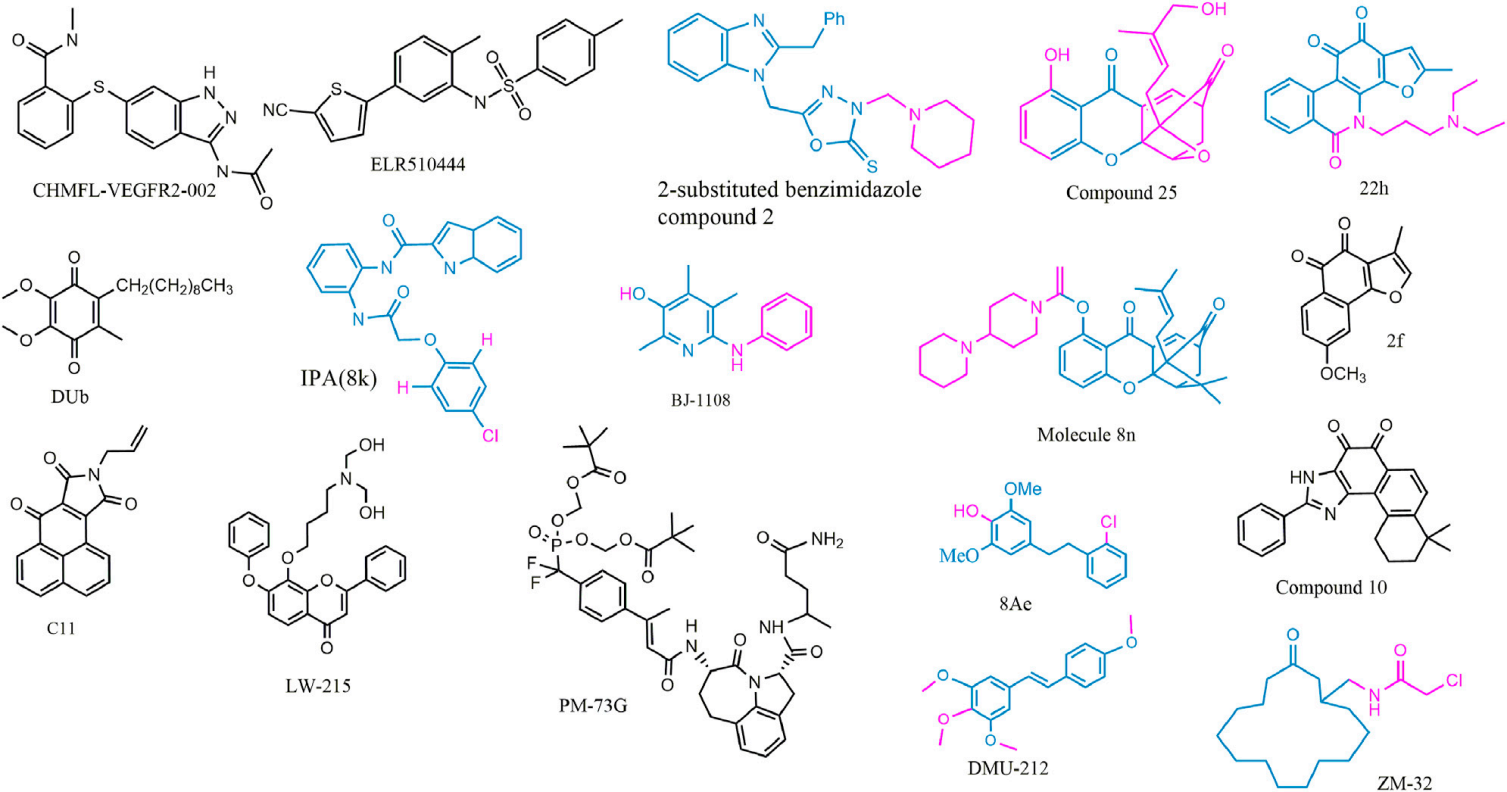
2D structure of potential chemicals with anti-angiogenesis. The structure–activity relationship in coloring molecules was investigated: blue (parent) and red (substituent position).

Based on the reported molecules with vascular regulatory activity, the development of new candidates is still the dominant method, while physiological molecular mimics and receptor blockers also deserve attention, as shown in Figure 4 and Table 2. These candidate molecules primarily consisted of physiological molecular mimics, heterocyclic compounds, flavonoids, anthrone, phenanthraquinones, and polyphenols. After a structure–activity relationship (SAR) investigation, the molecular candidates performed potent anti-cancer effects in tumor angiogenesis and improved pharmacokinetics properties, including but not limited to aqueous solubility and bioavailability.

**TABLE 2:** Anti-angiogenic effects and mechanisms of compounds related to ethnopharmacology.

Molecule	Model	Angiogenetic effect	Mechanism	Reference
CHMFL-VEGFR2-002	HUVECs and Zebrafish embryonic models	Inhibiting cell migration, invasion, and tube formation; intersegmental vessel (ISV) growth	VEGFR2 kinase ↓	Jiang et al. (2020a)
ELR510444	Renal cell carcinoma A498 and 786-O cells and xenograft tumor mice	Reducing VEGF release; inhibiting tumor angiogenesis	HIF-1α and HIF-2α ↓	Carew et al. (2012)
C11	Human microvascular endothelial cells (HMEC-1); CAM	Suppressing migration and tube formation; inhibiting angiogenesis *ex vivo*	FGFR1 and its downstream p-Akt and p-Erk ↓	Chen et al. (2019a)
PM-73G	MDA-MB-468 breast tumor xenograft mice	Reducing MVD	VEGF and p-STAT3 ↓	Auzenne et al. (2012)
BJ-1108	HUVECs and CAM	Inhibiting migration, tube formation, and angiogenesis *ex vivo*	Phosphorylation of PI3K, Akt, and mTOR ↓	Banskota et al. (2016)
DUb	CAM, YSM, and Matrigel plug assay; HUVECs	Reducing angiogenesis *in vivo* and *ex vivo*; inhibiting proliferation, migration, and tube formation	ROS/P53/BAI1 ↑	Cao et al. (2020)
IPA	HUVECs; CAM, rat aortic ring assay, and mice bearing Dalton’s lymphoma tumor	Inhibiting migration and tube formation; decreasing angiogenesis *in vivo* and *ex vivo*	P53↑; HIF-1α and its downstream VEGF and MMP-2,9 ↓	Al-Ostoot et al. (2021)
DMU-212	HUVECs; CAM and Matrigel plug assay	Inhibiting cell viability, migration, tube formation, and inducing apoptosis; reducing angiogenesis	Phosphorylation of VEGFR2 and its downstream c-Src, FAK, Erk1/2, Akt/mTOR/,and p70S6K ↓	Chen et al. (2013b)
LW-215	HUVECs; CAM, and rat aortic ring assay	Inhibiting migration and tube formation; reducing angiogenesis	Phosphorylation of VEGFR2, Akt, Erk 1/2, and P38 ↓	Zhao et al. (2018)
Compound 25	HUVECs	Inhibiting cell migration	HIF-1α ↓	Xu et al. (2016a)
Molecule 8n	HUVECs; fluorescent zebrafish assay (VEGFR2: GFP)	Suppressing migration, invasion, and tube formation; reducing angiogenesis *in vivo*	Hsp90/HIF-1α/VEGF in HepG2 cells ↓	Xu et al. (2016b)
2f	HUVECs; zebrafish embryo assay	Inhibiting proliferation, migration, and tube formation; reducing angiogenesis *in vivo*	Not reported	Huang et al. (2021)
8Ae	Zebrafish embryo assay	Reducing angiogenesis *in vivo*	Not reported	Guan et al. (2019)
ZM-32	HUVECs; xenograft mice models of MDA-MB-231 cells	Inhibiting migration and tube formation; reducing MVD	HuR, VEGF, and MMP-9 ↓	Yang et al. (2021a)

HUVECs, human umbilical vascular endothelial cells; CAM, chick chorioallantoic membrane; MVD, microvascular density; YSM, yolk sac membrane.

To avoid the side effects caused by poor selectivity of drugs against VEGFR, a novel inhibitor (CHMFL-VEGFR2-002) with high selectivity toward VEGFR-2 (inhibitory activity of kinase IC50 = 66 nmol/L) has been found to show superb anti-angiogenesis effect *in vivo* and *in vitro* with low toxicity (Jiang et al., 2020a). ELR510444, a small molecule blocking HIF and known as a microtubule blocker, inhibited tumor angiogenesis in mice model of renal cell carcinoma, which was contributed to the suppression of HIF-1α and HIF-1β activity (0-100 nM) and the induction of microtubule destabilization (EC50, 27 nM) (Carew et al., 2012). C11, an FGFR1 inhibitor, blocked cell migration and tube formation in HMEC-1 endothelial (1–10 uM) and angiogenesis in CAM assay (0.1 - 10 ng/egg) (Chen et al., 2019a). The JAK/STAT pathway enhances the progression of angiogenesis, which mainly relates that p-STAT3 responds to FGF2 and VEGF stimulated in tumors and ECs (Chen et al., 2008; Zhao et al., 2011).

STAT3 bound the VEGF promoter and transactivated VEGF to touch upon tumor angiogenesis (Niu et al., 2002). PM-73G, phosphopeptide molecular mimic that synthesized to target the SH2 domain of STAT3, reduced MVD and VEGF levels by the suppression of p-STAT3 (Tyr705) in mice bearing MDA-MB-468 tumor (Auzenne et al., 2012).

The 6-amino-2,4,5-trimethylpyridin-3-ol analogs had been investigated as feasible tumor angiogenesis regulators (Kim et al., 2014). BJ-1108, a 6-amino-2,4,5-trimethylpyridin-3-ol derivative, inhibited tumor angiogenesis and 5-HT-induced ROS generation that depended on the PI3K/Akt/NOX signaling pathway (0.1.1 μM) in HUVECs (Banskota et al., 2016). The decylubiquinone (DUb), a coenzyme Q analog, induced tumor angiogenesis inhibition of breast cancer *in vivo* and *ex vivo* by the ROS/p53/BAI1 signaling axis (Cao et al., 2020).

The new 2-substituted benzimidazole molecules with heterocyclic were synthesized, in which compound 2 had the best activity of anti-proliferation without genotoxicity in PC-3 and SK-BR-3 cancer cells (IC50 < 20 μg/ml) and anti-angiogenesis in CAM assay (Güner et al., 2019). The IPA(8k), a novel indolephenoxyacetamide analog with anti-proliferative activity against A549 (IC50 ∼5 uM), performed anti-angiogenic activity *in vivo* and *in vitro* through inhibiting HIF-1 alpha, VEGF, MMP-2 and -9, and P53 (Al-Ostoot et al., 2021).

DMU-212 (trans-3,4,5,4 ′- tetramethoxystilbene), a resveratrol analog with higher anti-tumor activity and bioavailability than resveratrol, exhibited effective inhibition toward angiogenesis *in vitro* and *in vivo*. In mechanism, it suppressed the VEFGR2/Akt/mTOR/p70S6K pathway and c-Src/FAK/Erk 1/2 axis (Chen et al., 2013b). LW-215, derived from flavonoid wogonin, attenuated tube formation by inactivating VEGFR2 and its downstream p-Akt, p-ERK1/2, and p-p38 in endothelial cells (Zhao et al., 2018).

The compound 25, a gambogic acid (GA) analog, inhibited the ATPase activity of Hsp90 with an IC_50_ value of 3.68 μM compared with GA 21.98 μM. In addition, the compound 25 suppressed migration and angiogenesis by downregulating HIF-1α that was regulated by Hsp90 in HUVEC cells (0.01-0.25 μM) (Xu et al., 2016a). Another investigation about GA derivatives implied molecule 8n that bears a strong resemblance to the aforementioned effects (Xu et al., 2016b).

The compound 22h obtained from tanshinone I elevated water-solubility, bioavailability and anti-tumor potency, while it also suppressed migration and tube formation of HMEC-1 cells (Ding et al., 2018; Tian et al., 2018). The molecule 2f, cleaved ring A of tanshinone IIA and imported a methoxy group at C-8 position, provided feasible physicochemical property and anti-angiogenic activity in HUVECs (0.25, 0.5, 1 μM) and zebrafish model (1,2,4 μM) (Huang et al., 2021). However, the eleven novel tanshinone analogs were obtained from puried tanshinone mixture from *Salvia miltiorrhiza* by one-pot synthesis modification, in which the molecule 10 exerted potent pro-angiogenesis effect in zebrafish, with at least partly involving VEGF/FGF-Src-MAPK and PI3K-P38 signaling pathways (Zhang et al., 2014).

The moscatilin derivative 8Ae performed more effective angiogenesis inhibition in zebrafish assay (0.62–1.25 μM) than positive drug SU5416 (Guan et al., 2019). Muscone derivative ZM-32 attenuated the stabilizing effect of RNA-binding protein HuR toward Vegf-a and Mmp9 mRNA, thus resulting in downregulation of VEGF-A and MMP-9 expression in HUVECs and breast cancer MDA-MB-231 cells (Yang et al., 2021a).

## Pharmaceutical Delivery Systems Against Tumor Angiogenesis

Nano preparations demonstrate the advantages of prolonging drug action time, improving solubility, and active and passive targeting (Hatami et al., 2020). Beyond loading anti-angiogenic drugs, nanoparticles are also combined with other adjuvants to construct multifunctional nano-platforms, including imaging, immunomodulation, photothermal therapy, and photodynamic therapy.

Galbanic acid (GC), delivered by the poly (D, l-lactide)–polyethylene glycol (PLA–PEG) nanosystem, showed lower IC_50_ than free galbanic acid in colon carcinoma C26 cells with IC_50_ = 8 μM and 15 μM, respectively, and increased by 15% in anti-angiogenetic activity compared to GC (Afsharzadeh et al., 2019). HA-TQ-Nps, hyaluronic acid-decorated mixed Pluronic® nanoparticles loading thymoquinone, faded tumor angiogenesis *via* miR-361/VEGF-A in breast cancer MDA-MB-231, MDA-MB-231, and 4T1 cells (IC50 < 9 μg/ml) (Bhattacharya et al., 2020; Peng et al., 2021). The amphiphilic and self-assemble drug (FUDR-PAB nanoparticles) was synthesized by conjugating floxuridine (FUDR) as the hydrophilic moiety and pseudolaric acid B (PAB) as the hydrophobic. The nanoparticle exerted better antiproliferative activity (lower IC_50_ and smaller tumor volume) in HeLa tumor cells and mice bearing tumors and higher anti-angiogenesis efficiency in HUVECs than PAB, FUDR, and PAB/FUDR mixture (Sun et al., 2019). Likewise, AuNPs-Qu-5, gold nanoparticle-loaded quercetin (50 μM), was observed to perform angiogenic nature against tube and new blood vessel formation *ex vivo* and *in vivo* through the VEGE/VEGFR2/PI3K/Akt axis, which was respectively confirmed by tube formation and CEA (Balakrishnan et al., 2016).

Cyclic RGD pentapeptide blocking α_v_β_3_ integrin and tengflavin were linked by low molecular weight heparin to form cRHG nanoparticles that inhibited HIF-1 α, VEGF, CD31, and p-VEGFR2 in U87MG glioblastoma xenograft model (Dahmani et al., 2016). RGD peptide surface-decorated selenium nanoparticles (RGD-NPs) loading with adriamycin significantly promoted the anti-angiogenic activity of SeNPs *in vitro* and *in vivo*. RGD-NPs induced apoptosis and S phase cell cycle arrest in HUVECs (2-8 μM) and inhibited neo-angiogenesis in BC MCF-7-bearing tumor mice (2.5–7.5 mg/kg) by the downregulation of VEGF-VEGFR2 (Fu et al., 2016). The spontaneous degradation of pH-degradable poly (vinyl alcohol) (PVA) microgel depended on pH in an acidic tumor microenvironment. The decoration of dopamine (DA) on PVA microgels partially contributed to tumor adhesion and retention, which was the origin of dopamine (DA)-functionalized PVA microgels (DMGs). DMGs@Bev /DTX, PVA microgel encapsulated bevacizumab (Bev) and docetaxel (DTX), facilitated anti-tumor activity in 4T1-Luc cells (48 h treatment IC50 < 10 μg/ml), and anti-angiogenesis in tumor-bearing BALB/c mice (Chen et al., 2020b). The microgels with tumor-targeting and pH-degradable for the combination scheme of Bev and DTX performed better chemotherapy enhancement and anti-angiogenesis than other controls (Chen et al., 2020b). The metal–organic framework nanosystem named as aMMTm, designed on the strategy of photodynamic therapy (PDT) and anti-angiogenesis, was packed with porphyrinic Zr-MOF (photosensitizer) and apatinib (VEGFR2 inhibitor) and coating with MnO2 and cell membrane in the surface (Min et al., 2019). PDT and VEGF/VEGFR double inhibitor (Avastin + Erbitux) lead to an obvious decrease in VEGF and EGFR and considerable tumor elimination in a murine bladder tumor model (Bhuvaneswari et al., 2011). Sunitinib, VEGFR and PDGFR inhibitor, was encapsulated in a polyamide amine (PAMAM) dendrimer cavity, and the nano-scintillator CaF2 and photosensitizer Rose Bengal were distributed on the surface of PAMAM at a suitable distance to construct a nano platform CCT-DPRS. After low dose X-ray irradiation, the doped scintillator transformed the captured energy into green emission, which led to further excitation of Rose Bengal to produce cytotoxic singlet oxygen to eliminate cancer cells. At the same time, the platform released sunitinib and active oxygen to induce apoptosis and inhibit tumor angiogenesis, with increased expression of cleaved PARP and decreased levels of VEGFA, HIF-1α, survivin, and p-STAT3 (Jiang et al., 2021).

Nanoparticles (CA4P-loaded NBP@TiO2) for photothermal therapy (PTT) combined with anti-angiogenesis have been reported, in which Au nanobipyramids (NBPs) were designed as photothermal agents for infrared light excitation at 1064nm and TiO_2_ shell coating with combretastatin A-4 phosphate (CA4P) for anti-tumor attribute. The inhibition of angiogenesis in HUVECs and reduction of tumor microvessel density in A549 tumor-bearing mice were found in the therapy with synergism between PTT and nano-platform (Chen et al., 2019b). Similar photothermal chemotherapy was applied in cervical cancer for anti-angiogenesis. The delivery nanosystem (cisplatin–AuNRs@SiO2–Avastin@PEI/AE105) carried cisplatin and the anti-angiogenic drug Avastin, with Au nanorods (AuNRs) selected as a photothermal agent. AE105, a polypeptide composed of nine molecular amino acids with a high affinity for uPAR receptor that is highly expressed in cervical cancer tissues, linked by hydrophilic polymers PEI to the nanoparticles for tissue targeting (Hu et al., 2019). RBCs@Se/Av suppressed angiogenesis of HUVEC by triggering apoptosis and decreased vascular density in A375 tumor-bearing mice. The nanosystem was constructed by binding pegylational selenium nanoparticles (SeNPs, Se) and Avastin (VEGF antibody, AV), encapsulated with red blood cells membrane (Liu et al., 2018b). A novel chelating agent, imidazole doped with organic silica (Imi-OSi) nano-materials, performed anti-angiogenesis by copper capture and blocking tumor blood vessels through phosphate and Cu^2^^+^ responsive polymerization in breast cancer and colon cancer mice models (Yang et al., 2019b).

Antagonizing cytokines triggering angiogenesis, combined with vascular normalization therapy, reshuffle the balance of pro-vascular factors and anti-vascular factors in TME. The bi-directional nanosystem has been exposed that FLG nanoparticles loaded with VEGF/VEGFR2 pathway inhibitors, low molecular weight heparin (LMWH), and gambogic acid (GA) and modified by F3 peptide targeting tumor vascular endothelial cells, obstructed the abnormal proliferation of vascular endothelial cells, increased pericyte coverage, and improved hypoxia, while the other nanosystem MAR/MPA with CCL5/CCR5 blocker Maraviroc induced the decrease of glycolysis rate, VEGF secretion, and Tregs recruitment as well as the increase of CD8 + T and CD4 + T cell infiltration (Deng et al., 2021).

The ginsenoside Rg3, oridonin, and Ganoderma lucidum polysaccharide (GLP) were introduced into a self-microemulsifying drug delivery system (RGO-SMEDDS) as an anti-angiogenic agent, immune regulator, and apoptosis inducer, respectively. The system evinced a combined strategy against HCC *via* triggering angiogenesis inhibition, anti-proliferation, and decreasing immunosuppressive cytokines and M2-polarized macrophages, for the suppression of the p-EGFR/AKT/GSK3 axis (He et al., 2021).

Sibusiso et al. have reported the nanosystem doped with artemisinins, including polymeric, metal-based, and lipid nanoparticles, against cancer by enhancing effects recuperating poor solubility and bioavailability and targeting delivery (Alven and Aderibigbe, 2020). Concurrently, Yun et al. depicted a biodegradable poly (ethylene glycol) methyl ether-poly (ε-caprolactone) (MPEG-PCL) loading dihydroartemisinin (DHA) had allowed for a stronger anti-angiogenic effect than free DHA (Lu et al., 2020).

## Co-Regulating Tumor Angiogenesis and TME by TCM

The molecular mechanisms through which immunosuppressive microenvironment-caused abnormal tumor vasculature and vascular normalization improved immunotherapy have been reported, respectively (Fukumura et al., 2018; Liu et al., 2021b). In brief, tumor-characteristic metabolism led to an imbalance between pro- and anti-angiogenic factors to induce abnormal vessels: disorganized vessel distribution and dysfunction, which in turn aggravated tissue hypoxia to promote the imbalance. The hypoxia, acidosis, and accumulated pro-angiogenic factors (represented by VEGF and Ang2) cooperatively promoted immunosuppressive TME with multi-mechanisms *via* recruiting immune-inhibiting cells and repressing the anti-tumor function of dendritic cells and cytotoxic T lymphocyte (Fukumura et al., 2018; Zhu et al., 2021b; Fousek et al., 2021). Also, on the contrary, the adverse TME recruited and activated immunosuppressive cells to facilitate tumor angiogenesis with the VEGF/VEGFR-dependent approach (Yang et al., 2021b).

Advances of angiogenesis dictated the efficiency of immunotherapy, while immune interference induced vascular normalization, which was ascribed to interferon γ secreted by CD4^+^ /CD8+ T cells, and reshaped TME advantageous to regression of angiogenesis in preclinical observations (Liu et al., 2021b; Yang et al., 2021b). The combined concept of immunocheckpoint blocking (PD-1/PD-L1 antibodies) and anti-VEGF has been generally accepted for the improvement of clinical outcomes and is considered a hopeful and promising treatment approach in therapeutics (Hack et al., 2020; Lee et al., 2020). Instead of directly targeting VEGF, an indirect approach was recommended for materializing dual-modulation of the tumor vascular system and TME through VEGF/HIF-1 mediated by the PI3K/Akt/mTOR cascade and preventing drug resistance (Fokas et al., 2012).

What we are persuasively interested in is the local or ethnic medicine—derived molecules with properties of both anti-angiogenesis and TME remodeling, all of them provide a unique sight on the combined therapy of tumor angiogenesis inhibition and immune modulation. On the other hand, there are abundant evidences implying that natural products and their analogs have a crucial part in regulating TME, such as ginseng and silibinin (Deep and Agarwal, 2013; Li et al., 2021b), while in independent studies the anti-angiogenic attributes in some of them are also reported. The biologically active ingredients with TME regulatory and anti-angiogenic activities confirmed in independent and respective studies are considered second-line or potential evidences. Traditional and empirical medicines, including traditional Chinese medicine, have contributed molecules libraries for cancer treatment and preclinical investigations with multi-potency in the modulating phenotype, such as angiogenesis and TME remodeling (He et al., 2015). The plant-derived flavonoids, alkaloids, glycosides, terpenoids, and coumarin were discussed as dispatchers between anti-VEGF therapy and treatment *via* immune checkpoint inhibitors (Kumar et al., 2021).

Except for the antiangiogeneic nature, as mentioned earlier, the immunomodulation of *Dolichos lablab* L. lectin was advantageous to anti-tumor effects mediated by IL-2 (Vigneshwaran et al., 2017). The digitoxin, famous as cardiotonic steroids, rescued HUVEC migration and loop formation that macrophages induced and also arrested SKOV3 cell growth and migration under macrophage conditioned media (Trenti et al., 2018; Whayne, 2018; Bejček et al., 2021). It also weakened HIF-1α protein expression by suppressing the phosphorylation of ribosomal protein S6 kinase (p70S6K) and eIF4E binding protein-1 (4E-BP1) in colon cancer cells (Mi et al., 2022). Significantly, luteolin enabled inhibition of angiogenesis induction of M2-like TAMs, achieved by the downregulation of HIF-1α and STAT3 signaling (Fang et al., 2018). Targeting macrophages in TME, anemoside A3 derived from Pulsatilla saponins induced tumor-suppressive M1-like macrophage by activating TLR4/NF-κB/MAPK signaling and subsequently enhanced expression of IL-12 in macrophages to attenuate angiogenesis of breast cancer *in vivo* and *in vitro* (Yin et al., 2021).

Bufalin, the bioactive C-24 steroids extracted from traditional Chinese medicine toad venom, exerted synergistic effects on angiogenesis with sorafenib *via* downregulating PI3K/Akt/mTOR/VEGF signaling pathways in HCC and HUVECs (Wang et al., 2016; Wang et al., 2018). Interestingly, bufalin inhibited angiogenesis mediated by TME cells (TAMs, CAFs, and CT26 cells) in the HUVECs model through regulating p-STAT3 and its downstream pro-angiogenic factors, including VEGF, PDGFA, E-selectin, and P-selectin (Fang et al., 2021).

Melittin (MEL), a polypeptide and the capital ingredient of honey bee venom, declined HIF-1α/VEGF levels *via* the suppression of ERK and mTOR/p70S6K signaling (Shin et al., 2013). It downregulated NF-κB to inhibit the HIF-1α/VEGFA and LDHA expression that caused angiogenesis and descent pH *via* anaerobic metabolism, in TME, respectively (Mir Hassani et al., 2021). CDDO-Me (Bardoxolone methyl), an analog of the natural triterpenoids oleanolic acid, the methyl-ester of the 2-cyano-3,12-dioxooleana-1,9 (11)-dien-28-oic acid (CDDO), have been abundantly advised for pharmacological applications, including tumor interference (Borella et al., 2019). CDDO-Me was reported to reduce chemokines CXCL12 and CCL2 release and the infiltration of suppressive TAM and inhibit cyclin D1, EGFR, and STAT3 responsible for anti-proliferation in PyMT breast cancer (Tran et al., 2012). Another study in PyMT implied that CDDO-Me decreased IL-10 and VEGF levels while increased TNF expression, concomitantly suppressed TAM tumor infiltration, and CD4 Foxp3 regulatory T cells (Ball et al., 2020). The nanoparticle delivery of CDDO-Me reshaped the immunosuppressive microenvironment with abatement of both Treg cells and MDSCs, and the concurrent rise of cytotoxic T-lymphocyte population, meanwhile, reduced angiogenesis in B16F10 melanoma mice (Zhao et al., 2015). The nanoparticle delivery of anti-tumor agent silibinin and PI3Kγ blocker IPI-549 synergistically remodeled TME in 4T1 breast cancer mice, contributing to decline of TAFs, MDSCs, tumor angiogenesis, and matrix but increased Treg cells (Jiang et al., 2020b).

The nanosystem delivery technology was considered a promising means for reshaping abnormal tumor vasculature to vascular normalization (Liang et al., 2022). On the other hand, vessel normalization was beneficial to the enhancement of drug penetration (Li et al., 2020b). Another strategy for co-targeting angiogenesis and TME is chosen as delivery platforms with functional decorations, in which loaded two or more molecules or drugs achieved the aforementioned goals with a designed enhancement of pharmacokinetics, while nanosystems loading single candidates are also that we are interested in. The normalization of blood vessels based on the scheme of angio-blocker delivery has been reported to reprogram TME for improving immune cell infiltration (Li et al., 2020b). For instance, the nomoplatform based on anti-angiogenic low molecular weight heparin (LMWH) transformed M2 polarized macrophage to M1 type and induced the vessel normalization (Xu et al., 2020). Pleiotropic nanodelivery was vastly commendable to co-modulate suppressive TME and tumor angiogenesis with multiple components, and the evidence entailed poly-lactic-glycolic acid (PLGA) and liposome nanoparticles (Hameed et al., 2018). The abundant multi-potency nanosystems indicated a synergistic strategy by introducing chemotherapy and dynamic therapy (sonodynamic and photothermal dynamics) for anti-angiogenesis (Li et al., 2020b; Zhu et al., 2021b). We expect that a multi-strategy delivery system and single molecules with multi-efficiency emerge to illuminate unlisted angiogenesis blockers for reshaping suppressive TME, while the role of the classic recipe of TCM remains underlying.

## Perspectives and Conclusion

Tumor angiogenesis is one of the crucial factors that shape cancer malignancy. The design and development of anti-angiogenic drugs, the identification of potential regulatory networks, and further application of delivery systems are beneficial to provide progressive insights into tumor angiogenesis. Some TCMs or bioactive molecules are typical with the VEGF/VEGFR-dependent approach against tumor angiogenesis, such as *Salvia miltiorrhiza*, *Curcuma longa*, ginsenosides, and *Scutellaria baicalensis* (Zhang et al., 2018a). Several bioactive ingredients or phytomolecules from the ethnopharmacology we mentioned, mainly including artemisinin, tanshinone, flavonoids, and saponin, are a miniature to depict and generalize these molecules’ attributes against tumor angiogenesis and associated regulatory mechanisms. Specifically, whatever natural products or chemical derivatives modulating angiogenesis share cardinal pathways that VEGF mediated, such as VEGFR2 pathway in vascular endothelial cells, HIF-1α/VEGF and its upstream transcriptional signaling in cancer cells, we concern and await the emergence of more potential targets for anti-angiogenesis therapy. Although other natural molecules in TCM also performed anti-angiogenesis property, comprising coumarins (Wu et al., 2020), terpenoids (Kamran et al., 2022), polysaccharides (Li et al., 2021c), and polyphenols (Li et al., 2021c; Marrero et al., 2022), they have no distinct difference in mechanisms in comparison with molecules we reported. Recently, angiopoietin (for instance, ang2) inhibition has been implied to be a strategy for overcoming the resistance of VEGF blockers in clinical, while the potential of natural molecules as angiopoietin inhibitors needs further observation (Parmar and Apte, 2021).

In spite of the fact that great understanding and progress have been achieved in angiogenesis modulation *via* molecules, such as TKIs in clinical and preclinical candidates, there is a massive dearth of investigation in ethnopharmacology or empirical medicine for comprehensively screening and proving potential angio-inhibitor from the natural molecule library, including TCM. Moreover, thyroid hormone induced angiogenesis through activation of αvβ3 integrin signaling and upregulation of VEGF, which suggests the potential value of endocrine therapy in anti-angiogenesis (Cayrol et al., 2019). What is exciting is that some active ingredients from TCM need to be relocated in angiogenic effects for the shared signaling pathways (such as MAPK and PI3K/Akt/mTOR), implying the viability of co-targeting angiogenesis and other phenotypes, including proliferation, apoptosis, and stem cell like-type. However, there are few studies to demonstrate the role of candidate molecules toward antiangiogenic factors or signalings, such as TNFα, TSP-1, TIMP, and TGF-β/BMP pathway (Ayuso-Íñigo et al., 2021), but IL-8 seems to involve the crosstalk between angiogenesis and TME like VEGF (Fousek et al., 2021). In clinical, these candidate drugs were more considered dietary supplements or adjuvants (Table 3) for the anti-angiogenic strategy, beyond a single drug being used. Thus, it is expected that more botanical or ethnic medicine-related molecules will be pushed into the clinic for anti-tumor and even based on the antagonism of tumor angiogenesis. Given vascular structure distortion and vascular dysfunction are conducive to tumor deterioration and metastasis, tumor vascular normalization and tumor microenvironment reprograming that the candidate molecules triggering may contribute to the progression in clinical and preclinical investigations.

**TABLE 3:** Clinical trials of molecules based on anti-angiogenic therapy against cancer in ethnopharmacology. Data from ClinicalTrials and WHOICTRP.

Nct code	Cancer	Drug	Phase	Start date	End date
NCT02439385	Colorectal Cancer	Avastin and curcumin	Phase 2	Aug 2015	Mar 2022
NCT02146118	Carcinoma and non-small-cell lung	Erlotinib and silybin-phytosome	Phase 2	Apr 2014	May 2014
NCT00529113	Pancreatic cancer	Bardoxolone methyl and gemcitabine	Phase 1	Sep 2007	Feb 2022
NCT00274820	Chronicmyeloproliferative disorders and leukemia	Ascorbic acid, arsenic trioxide, dexamethasone, and thalidomide	Phase 2	Oct 2005	Jul 2020

Single-cell transcriptome profiling suggests that SQLE and ALDH18A1 may be potential anti-angiogenic targets for modulating epithelial cell metabolism (Rohlenova et al., 2020). Interested in solid tumors based on single-cell transcriptomics, HIF-1α was reported to mediate the functional inhibition of NK cells and regarded as an immune checkpoint of NK cells, which opened novel insights into regulating the crosstalk between angiogenesis and TME *via* HIF-1α blocking (Ni et al., 2020). Although single-cell RNA sequencing is emerging, it still needs to be further observed for evaluating angiogenesis. However, there is no report concerning tumor angiogenesis in spatial transcriptomics (ST). Interestingly, it can be considered a supplementary method to observe multiple angiogenesis markers on tissue sections for annotation in RNA expression, while the GeoMx Digital Spatial Profiler technology has integrated the transcriptomics and proteome.

While targeting VEGF and /or HIF-1 remains a basic starting point for verifying potential anti-angiogenic candidates and creating a bridge to interact with TME modulation, some novel strategies are imported into ethnopharmacology, composed of vessel normalization for the crosstalk between tumor angiogenesis and TME reconstruction. It should not be ignored that the cardinal role of angiogenic closely related tumor cells and endothelial cells in TME but reshaping tumor vessel *via* TAMs and macrophages, even fibroblasts, takes more attracted attention. Traditional Chinese medicine and bioactive molecules have been depicted as regulators in the crosstalk between gut microbiota and TME, yet the indirect involvement of TCM toward tumor angiogenesis *via* modulating the aforementioned crosstalk remains little understood (Wang et al., 2021). There are still challenges in the anti-angiogenic research of bio-active components of herbal or botanical extracts (Heinrich et al., 2020). The limitation of this article is that it just selectively elucidates the potential and representative regulators and delivery systems of tumoral angiogenesis, and there is a lack of view of the interaction of rather new but not fully explored non-coding RNAs and metabolic reprogram toward angiogenesis. Taken together, potential targets in vascular endothelial cells (ECs) and in tumor cells may provide new perspectives on angiogenesis, including CCN4, CCR5, ILT-3, EDD, and PIN. What should be paid attention to was that PFKFB3 played the mediator between glycometabolism and VEGF signaling in ECs. Plant-derived and ethnopharmacology-related molecules contributed to the anti-angiogenesis therapy *via* modulating VEGFR2 and its downstream PI3K/AKT/mTOR and MAPKs, with NF-κB, STAT3 and β-catenin involved, irrespective of targets in endothelial cells or cancer cells. The molecule library of natural products has not been fully explored in angio-modulation. Two methods were applied for developing anti-angiogenic drugs in medical chemistry: physiological molecular simulant and derivatives of natural products, in which the latter was featured by heterocyclic compounds, flavonoids, anthrone, phenanthraquinones, and polyphenols. Tanshinone’s analog has been extensively investigated as a potential anti-angiogenic agent. Moreover, nano-delivery systems loading functional molecules exerted an approach for anti-angiogenic therapy, even inducing tumor vessel normalization and remodeling TME simultaneously. Candidates with multi-potency, including digitoxin, bufalin, melittin, and CDDO-Me, make co-modulating therapy between tumor angiogenesis and immunosuppressive TME true in ethnopharmacology. We look forward to more clinical trials focusing on the anti-angiogenic and immunomodulatory properties of molecules from ethnopharmacology.
